# Biomarkers of HIV-1 associated dementia: proteomic investigation of sera

**DOI:** 10.1186/1477-5956-7-8

**Published:** 2009-03-17

**Authors:** Jayme Wiederin, Wojciech Rozek, Fenghai Duan, Pawel Ciborowski

**Affiliations:** 1 Department of Pharmacology and Experimental Neuroscience, University of Nebraska Medical Center, Omaha, NE 68198-5800, USA; 2 Department of Virology, National Veterinary Research Institute, 24-100 Pulawy, Poland; 3 Center for Statistical Sciences, Brown University, Providence, RI 02912, USA

## Abstract

**Background:**

New, more sensitive and specific biomarkers are needed to support other means of clinical diagnosis of neurodegenerative disorders. Proteomics technology is widely used in discovering new biomarkers. There are several difficulties with in-depth analysis of human plasma/serum, including that there is no one proteomic platform that can offer complete identification of differences in proteomic profiles. Another set of problems is associated with heterogeneity of human samples in addition intrinsic variability associated with every step of proteomic investigation. Validation is the very last step of proteomic investigation and it is very often difficult to validate potential biomarker with desired sensitivity and specificity. Even though it may be possible to validate a differentially expressed protein, it may not necessarily prove to be a valid diagnostic biomarker.

**Results:**

In the current study we report results of proteomic analysis of sera from HIV-infected individuals with or without cognitive impairment. Application of SELDI-TOF analysis followed by weak cation exchange chromatography and 1-dimensional electrophoresis led to discovery of gelsolin and prealbumin as differentially expressed proteins which were not detected in this cohort of samples when previously investigated by 2-dimensional electrophoresis with Difference Gel Electrophoresis technology.

**Conclusion:**

Validation using western-blot analysis led us to conclude that relative change of the levels of these proteins in one patient during a timeframe might be more informative, sensitive and specific than application of average level estimated based on an even larger cohort of patients.

## Background

HIV-1 penetrates the brain shortly after infection and remains there throughout entire disease. Approximately 50% of infected individuals develop some form of cognitive impairment ranging from an asymptomatic form diagnosed during formal testing to the most severe HIV-associated dementia (HAD) leading to death [1]. Although antiretroviral therapy (ART) has a profound effect on slowing disease progression, increasing survival and decreasing the number of HAD incidents from 30 to 7%, the rate of HIV-1 infected patients with HIV-associated Neurocognitive Disorders (HAND) remains the same [1,2]. In consequence, the prevalence of HAD has increased due to increased survival of these individuals [3-7]. These epidemiological data suggest that ART provides only partial protection against neurological damage in HIV-infected people [8].

Despite of more than 20 years of research efforts we are lacking good biomarkers supporting diagnosis of HAND including its most severe form, HAD [9,10]. Current diagnosis and identification of HAND is based on neuropsychological tests and exclusion of other potential causes such as opportunistic infections, tumor etc [11]. Laboratory tests of disease progression, although valuable, are not diagnostic and pose a need for more accurate and reliable markers to monitor progression of cognitive impairment [12-14]. Good and reliable diagnostic biomarkers are also indispensible for development of new therapeutic strategies. Discovery of biomarkers, which could be used to predict dementia and monitor disease progression, is important for the development of early and effective treatments designed to maintain normal cognition and quality of life [15,16].

Despite the technological progress in recent years in sample preparation for proteomic analyses, fractionation techniques and increased sensitivity of mass spectrometers, proteomic analysis of serum/plasma and cerebrospinal fluid (CSF) poses significant challenges [17-21]. High complexity and high dynamic range of proteins and peptides circulating in plasma and low levels of proteins originating from tissue leakage are just few of the most important challenges [22,23]. Immunodepletion of most abundant proteins from plasma/serum and CSF samples is the most common first step in reducing complexity of these samples. Although such approach has proven to be useful, further steps of sample fractionation are desirable [24].

Global proteomic profiling of clinical samples brought high expectations for accelerated discovery of new biomarkers to aid physicians in diagnosing and researchers in understanding molecular mechanisms of diseases. However, high dynamic range of plasma/serum and CSF proteins created challenges in such analyses. Immunodepletion became a standard first step, yet there is no consensus to how many of the most abundant proteins need to be removed. We have used IgY based technology for immunodepletion of CSF and sera samples in our previous studies [25,26]. Another challenge is the choice of a single or combination of profiling technology platforms. In our previous studies we used 2-dimensional electrophoresis (2DE) with Differential Gel Electrophoresis (DIGE) profiling method of immunodepleted CSF or sera from HIV-1 infected individuals with or without HAD [25,27] and demonstrated several differentially expressed proteins which can be potential biomarkers.

Although CSF surrounding the brain and spinal cord seems to be the best clinical material to reflect ongoing pathological processes [28-32], evaluations of the CSF proteome pose a challenge of availability of sufficient amount of protein in addition to high dynamic range of proteins. Alternatively, plasma or serum samples can be used. However, the question that remains is how closely changes in proteome profile of blood proteins reflect changes in the brain which is behind blood brain barrier (BBB). Because BBB is compromised during HIV infection, we posit that proteins from CSF leak into the blood and can be detected as biomarkers. In addition we expect that the plasma/serum proteome can be reflective of increased neuroinflammation. Although such surrogate biomarkers are not exclusive for HIV infection, they can be relevant and helpful as auxiliary tests [33].

Our current work indicates that multiple protein profiling approaches as well as multiple sample fractionation schemes are required to more completely assess changes in proteomes due to pathological changes [34]. Our data also indicates that biomarker levels should be measured relative to baseline for any individual to assess relative changes rather than comparing to a set threshold. Although putative biomarkers discovered during this study are unlikely to be stand alone measures of disease or suspected disease, they can be part of a broader diagnostic approach including psychological and brain imaging tests [35,36].

## Results and discussion

### Proteomic profiling

Serum samples used in this study were provided by NeuroAIDS Tissue Consortium and were obtained from HIV-1 infected individuals with or without HAD. Although introduction of ART resulted in significant decrease of HAD cases [1], we decided to use samples representing opposite spectrum of the disease to maximize chance in discovering biomarkers of an ongoing neurodegenerative process. Samples were immunodepleted from 12 most abundant proteins prior to proteomics profiling. For this purpose we used immunoaffinity chromatography with a column that is based on IgY technology [25,26]. In our previous proteomic investigation of this set of sera samples we used 2DE DIGE as profiling method and found three differentially expressed proteins: complement C3, ceruloplasmin and afamin [26]. Differential expression of two of them, ceruloplasmin and afamin, was further validated by western blot analysis [26].

**Figure 1:**
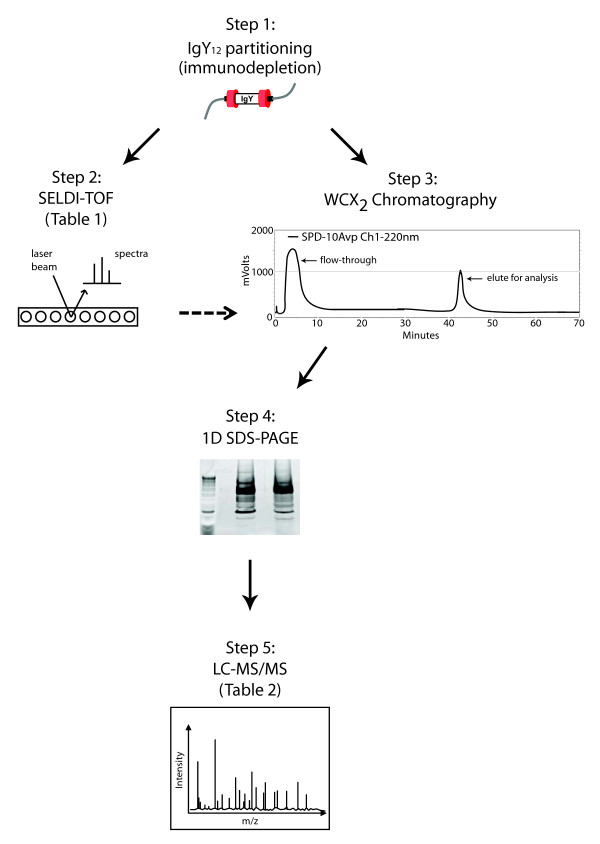
**Experimental design**. Flow chart presenting experimental design of proteomic analysis combining immunodepletion, SELDI-TOF, ion exchange chromatography, 1D SDS-PAGE and LC MS/MS.

In this study we used SELDI-TOF ProteinChip^® ^assays to profile proteomes of the same immunodepleted serum samples as in previous published study. We hypothesized that by applying different profiling approach we will discover differentially expressed proteins which were not found using 2DE DIGE. We choose SELDI-TOF profiling because of two reasons. One was to investigate proteins and/or their processed forms in low (~4 kDa) to medium low (~28 kDa) range of molecular weight for which 2DE DIGE is not an optimal profiling method. Our second reason for using SELDI-TOF ProteinChip^® ^technology was an ability of direct translation of chromatographic conditions from analytical to preparative mode as indicated as steps 3 and 4 in Figure 1. We have previously used this approach for identification of differentially expressed proteins [37]. Acquired SELDI-TOF spectra were subjected to rigorous statistical analysis and resulted in the identification of 50 peaks which potentially represent differentially expressed proteins. Among those 50 peaks detected, 21 showed statistically significant differences in intensities (p < 0.05) (Additional file 1, Table S1), however only 4 showed high significance, as illustrated in Table 1. Two peaks with m/z 4,493 and 25,872 showed increased intensity associated with HAD and 2 other peaks showed opposite trend (Table 1). Considering the highly variable nature of SELDI-TOF spectra we approached interpretation of such spectra with caution, therefore, some differences showing borderline significance may not be confirmed when larger number of spectra is generated.

**Table 1:** SELDI-TOF protein peaks in HIV-affected individuals with HAD compared to without HAD

Peak Number	Peak Median m/z	P-value^1^	Peak Intensity ^2^
1	4,493	0.000368	Increased

2	6,435	0.000196	Decreased

3	6,633	7.80E-05	Decreased

4	25,872	3.79E-06	Increased

^1) ^P value ≤ 0.001 (i.e., 0.05/50), the adjusted criteria after Bonferroni correction for multiple testing). ^2) ^Peak intensity of HIV-affected individuals with HAD as compared to without HAD.

### Weak Cation Exchange (WCX) chromatography and 1DE

**Figure 2:**
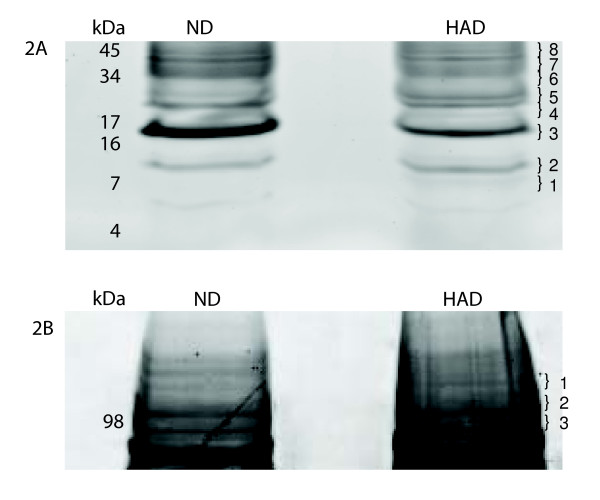
**1D Electrophoresis**. 1D SDS-PAGE. ND and HAD pooled samples were first depleted from 12 most abundant proteins, then processed through a weak cation exchange column, then 20 ug of each sample was loaded on 16% tricine gel and stained with sypro ruby. Numbers on the right-hand side of gels indicate band number that corresponds to Table 2 and 3, respectively. ND-non-demented; HAD-demented.

First dimension fractionation was immunodepletion of 12 most abundant proteins using IgY12 immunoaffinty chromatography. Therefore, preparative WCX chromatography was the second dimension fractionation reflecting conditions used in SELDI-TOF profiling. At the same time it allowed us to obtain amounts of protein sufficient for one more step of sample fractionation and mass spectrometry based protein identification. Despite of two steps of fractionation, samples were still complex enough to compromise detection of differentially expressed proteins. Therefore, we used 1DE in the subsequent step. Because we were specifically interested in low molecular weight proteins we used 16% Tricine gel and developed protein resolution only to the half of a distance. This approach allowed us to separate proteins in the region from 4 to 28 kDa while maintaining undiffused protein bands (Figure 2A, boxes 1 to 8). As expected we observed differences in intensities of protein bands based on staining with SyproRuby reflecting differences in relative abundance of proteins. The most profound difference was observed in band 3 (Figure 2) and as illustrated in Figure 2 we excised corresponding gel cubes which were further subjected to trypsin digestion and subsequent protein identification based on nano-LC-MS/MS analysis. Table 2 summarizes identified proteins (corresponds to Figure 2A).

**Table 2:** Identifications of proteins in eight gel sections shown in Figure 2A

**Band #**	**Protein Name**	**MW**	**Swiss Prot**	**NCBI**	**Peptides**	
					**ND**	**HAD**

2	pro-platelet basic protein precursor	13894.1		4505981	2	0

2	unnamed protein product	66019.2	B2RA01	189054178	2	2

						

3	prealbumin	15919.0	P02766	219978	5	0

3	unnamed protein product	66019.2	B2RA01	189054178	2	0

						

4	unnamed protein product	66019.2	B2RA01	189054178	2	0

4	prealbumin	15919.0	P02766	219978	2	2

4	TETN_HUMAN Tetranectin precursor	22566.7	P05452	267108	3	2

4	Complement component C8 gamma chain	22219.3	P07360	29577	0	2

						

5	A Chain A, Crystal Structure Of Human Apolipoprotein A-I	23403.4		2914175	0	2

5	apolipoprotein A-I preproprotein	30777.6		4557321	0	2

5	TETN_HUMAN Tetranectin precursor	22566.7	P05452	267108	0	2

5	L Chain L, Crystal Structure Of Tissue Factor In Complex With Humanized Fab D3h44	23499.0		18655500	0	2

						

6	mannose-binding lectin	26090.3	P11226	5911809	2	2

6	inter-alpha-trypsin inhibitor family heavy chain-related protein	103384.8	Q14624	1483187	2	2

6	apolipoprotein A-I preproprotein	30777.6		4557321	0	2

6	A Chain A, Crystal Structure Of Human Apolipoprotein A-I	23403.4		2914175	0	2

6	L Chain L, Crystal Structure Of Tissue Factor In Complex With Humanized Fab D3h44	23499.0		18655500	0	2

						

7	inter-alpha-trypsin inhibitor family heavy chain-related protein	103384.8	Q14624	1483187	3	3

7	complement factor I preproprotein	65720.0		119392081	3	0

7	mannose-binding lectin	26090.3	P11226	5911809	2	2

7	B Chain B, Human Complement Component C3	112940.1		78101268	4	0

7	clusterin isoform 2	52494.3		42740907	2	0

						

8	complement factor I preproprotein	65720.0		119392081	0	3

8	clusterin isoform 2	52494.3		42740907	0	2

8	inter-alpha-trypsin inhibitor family heavy chain-related protein	103384.8	Q14624	1483187	0	2

The most profound difference observed in band 3 of Figure 2A was also reflected by the degree of sequence protein coverage of identified prealbumin (NCBI accession# BAA00059) which is now called transthyretin. The only difference in amino acid sequence of 147 a.a. long polypeptide chain of prealbumin and 148 a.a. long transthyretin (NCBI accession# AAP36542) is one additional Leu as C-terminal residue in the latter protein. Electrophoretic migration of this protein corresponds well with its theoretical molecular weight of prealbumin 15,919 Da as well as transthyretin 16,032 Da (Figure 2A).

In the subsequent experiment we used 4–12% Bis-Tris gel to separate proteins in the high molecular weight region (Figure 2B). We did not investigate proteins of this size using SELDI-TOF profiling. Our previous experience with this technology platform showed that while in a complex mixture very few proteins above 50,000 m/z mark can be detected using our criteria of the first and second pass. Such proteins were usually detected as broad peaks with low intensity and relatively high difference between theoretical and experimentally measured masses. Nevertheless, we predicted that the applied scheme of fractionation and profiling will result in uncovering differential expressed proteins in high molecular weight range. Results of 1DE analysis are shown in Table 3 (corresponds to Figure 2B). We observed one protein band which showed clear difference in abundance and was higher in HAD. Subsequent LC-MS/MS analysis of tryptic digest resulted in identification of gelsolin (Accession# gi|121116|sp|P06396.1|GELS_HUMAN [121116]).

**Table 3:** Identifications of proteins in three gel sections shown in Figure 2B

**Band #**	**Protein Name**	**MW**	**Swiss Prot**	**NCBI**	**Peptides**	
					**ND**	**HAD**

1	vitamin D-binding protein/group specific component	52950.4	P02774	455970	3	5

1	CO4A_HUMAN Complement C4-A precursor (Acidic complement C4)	192769.5	P0C0L4	81175238	2	2

1	complement factor B preproprotein	85532.4	P00751	67782358	3	5

1	afamin precursor	69068.8	P43652	4501987	3	2

1	inter-alpha (globulin) inhibitor H1	101401.4		4504781	7	4

1	complement component 1 inhibitor precursor	55153.9	P05155	73858568	1	2

1	attractin isoform 1	158535.6	O75882	21450861	2	1

1	complement factor H	51033.7	P08603	758073	1	1

1	factor H	139124.7		31965		1

1	alpha 1B-glycoprotein	54253.2	Q68CK0	21071030	1	2

1	Complement component 6 precursor	104843.6	P13671	4559406	2	3

1	ITIH2_HUMAN Inter-alpha-trypsin inhibitor heavy chain H2 precursor (ITI heavy c	106435.6	P19823	125000	2	2

1	A Chain A, Complex Of The Catalytic Domain Of Human Plasmin And Streptokinase	27286.2		5821850	1	3

1	serpin peptidase inhibitor, clade A, member 3 precursor [Homo sapiens]	47650.6		50659080	1	2

1	gelsolin isoform a precursor	85696.9	P06396	4504165	0	3

1	A2MG_HUMAN Alpha-2-macroglobulin precursor (Alpha-2-M)	163276.5	P01023	112911	7	0

						

2	proceruloplasmin	108339.1	P00450	219549	1	1

2	hemopexin	51676.1	P02790	11321561	2	2

2	afamin precursor	69068.8	P43652	4501987	2	2

2	vitamin D-binding protein/group specific component	52950.4	P02774	455970	2	7

2	CO4A_HUMAN Complement C4-A precursor (Acidic complement C4) [Contains: Comple	192769.5	P0C0L4	81175238	2	2

2	gelsolin isoform a precursor	85696.9	P06396	4504165	0	2

2	complement factor B preproprotein	85532.4	P00751	67782358	3	4

2	serpin peptidase inhibitor, clade A, member 3 precursor	47650.6		50659080	3	3

2	alpha 1B-glycoprotein	54253.2	Q68CK0	21071030	4	3

2	antithrombin III variant	52691.3	Q7KZ97	576554	2	3

2	complement component 1 inhibitor precursor	55153.9	P05155	73858568	2	3

2	ceruloplasmin precursor	122204.3	P00450	4557485	3	3

2	Complement component 6 precursor	104843.6	P13671	4559406	2	3

2	A Chain A, Complex Of The Catalytic Domain Of Human Plasmin And Streptokinase	27286.2		5821850	2	3

2	inter-alpha (globulin) inhibitor H1	101401.4		4504781	2	1

2	alpha-2-HS-glycoprotein	39324.4		4502005	1	2

						

3	CO4A_HUMAN Complement C4-A precursor (Acidic complement C4) [Contains: Comple	192769.5	P0C0L4	81175238	1	2

3	hemopexin	51676.1	P02790	11321561	2	2

3	complement factor B preproprotein	85532.4	P00751	67782358	1	6

3	vitamin D-binding protein/group specific component	52950.4	P02774	455970	5	7

3	Complement component 6 precursor	104843.6	P13671	4559406	4	4

3	complement component 2 precursor	83267.4	P06681	14550407	0	2

3	inter-alpha (globulin) inhibitor H1	101401.4		4504781	2	2

3	A Chain A, Complex Of The Catalytic Domain Of Human Plasmin And Streptokinase	27286.2		5821850	1	3

3	antithrombin III variant	52691.3	Q7KZ97	576554	1	2

3	complement component 1 inhibitor precursor	55153.9	P05155	73858568	4	3

3	plasminogen	90568.6	P00747	4505881	0	2

3	alpha-1-microglobulin; bikunin	16541.7		579676	1	2

### Western blot validation

Validation of putative biomarkers resulting from proteomic analyses poses a significant challenge. One of the reasons is high diversity in the levels of proteins within normal human population. For example, in CSF concentration of gelsolin ranges from 1.2 to 15.9 μg/ml [38].

**Figure 3:**
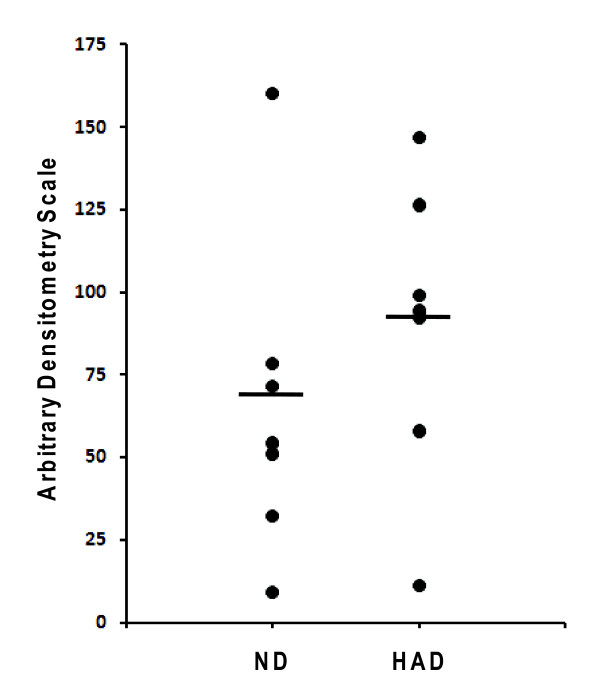
**Western blot**. Levels of gelsolin in sera samples from HIV infected patients with or without dementia. Arbitrary units are based on quantitative Western blot analysis. ND-non-demented; HAD-demented.

Among validation methods available at this time we have chosen western blot analysis [25,26]. This experimental approach is convenient because of relative high throughput and widely available software for quantitative analysis. Another advantage is small amount material required for such analysis which is important when quantity of sample is limited. Although usually serum/plasma is one of the most abundant clinical materials available for proteomic analysis, total pool of proteins obtained after immunodepletion of 12 most abundant proteins contains approximately 4–5% of the initial amount [22]. In this step, we used western blot combined with quantitative densitometry measurements of resulting bands to analyze levels of gelsolin in all 14 samples individually. Results presented in Figure 3 show a trend in increased expression of gelsolin in sera samples of patients with HAD; however, no statistically significant difference has been found between these two populations of samples. Lack of significant difference can be attributed to small sample number and the presence of outliers. We further hypothesize that because baseline level of gelsolin within normal human population is substantially variable, less profound changes due to pathological conditions when averaged may not yield statistical significance. Therefore, change observed for any given patient over time might be much more informative. This, however, needs to be investigated in a longitudinal study using samples from three groups of patients: one, those who showed signs of progression of cognitive impairment, two, those who showed signs of no change and three, and those who showed signs of improvement.

## Conclusion

**Figure 4:**
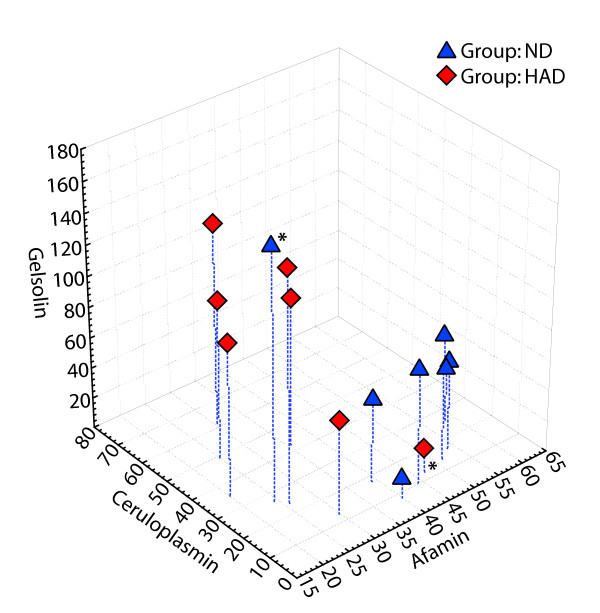
**Sample classification**. Classification of ND and HAD samples based on levels of afamin, ceruloplasmin and gelsolin as determined by densitometry measurements of Western blot films. ND-non-demented; HAD-demented.

It has been previously shown that one proteomic platform and/or profiling approach does not uncover all existing differences which can be valuable biomarker candidates [24,39-41]. This is also true for current investigation. Therefore, in this report we present continuation of our previously published profiling of sera from HIV-1 infected individuals with or without CI [26]. We used the same cohort of samples, however, different profiling approach and now found that gelsolin and prealbumin were differentially expressed. Previous approach using 2DE DIGE did not show differences in expression of these two proteins. This could be attributed to the fact that both proteins were located in high and low regions of gels used in 2DE, where resolution of spot is less favorable. Validation of our data using western-blot analysis did not reach statistical significance difference between average values because of high variability of expression of these two proteins within population. There were also few samples which we classified as outliers. If these samples are removed from analysis, statistical significance can be achieved. It has to be noted that the only criterion for sample classification was clinical diagnosis of HAD or lack of it. The far high or far low levels of proteins (biomarkers) might be attributed to other factors such as opportunistic infections etc. Nevertheless, addition of gelsolin as a biomarker candidate to ceruloplasmin and afamin as previously reported by us [26], reinforced our classification of HAD and non-HAD samples. Figure 4 represents combined results from previous [26] and current study. Another conclusion that we can make based on this study is that for future experiments cohorts of samples need to be assembled based on very careful clinical diagnosis of patients to eliminate variation introduced by other concurrent pathologies, e.g. HCV. This, in turn may reduce variability within the groups.

Summarizing, our main conclusion from this study is that because of high diversity of expression of some proteins among individuals, the relative change over time intervals such as treatment for any given patient might serve as more useful biomarker to aid other means of diagnosis of cognitive impairment than calculated average levels among large cohorts which will not generate statistically significant differences. HAND is a relatively slowly progressing disease therefore changes measured in weeks or even months intervals can still be useful as aid in diagnostics.

## Methods

For this study, we used 14 sera samples from HIV-1 infected individuals with (HAD) or without (ND) HIV-1-associated dementia (7 from each group). Samples were previously obtained from the National NeuroAIDS Tissue Consortium (NNTC, ) under Request# R101. Use of sera samples in this study has been approved by UNMC Institutional Review Board (#196-05-EX).

### IgY PARTITIONING (IMMUNODEPLETION)

We used the ProteomeLab IgY-12 High Capacity Proteome Partitioning Kit (Beckman Coulter, Fullerton, CA) to remove the 12 most abundant serum proteins, including albumin, fibrinogen, transferrin, haptoglobin, IgG, IgA, IgM, apo A-I and II, α1-antitrypsin, α1-acid glycoprotein and α2-macroglobulin. The kit included a HPLC affinity column LC10 (12.7 × 79.0 mm) with a capacity of 0.25 mL of human serum per cycle and optimized buffers for sample loading, washing, eluting and regenerating. Serum samples were centrifuged at 18,000 × g for 15 min at 4°C to pre-clear and delipidate samples prior to chromatography. The middle layer of pre-cleared serum was collected, diluted (0.25 mL of serum and 0.375 ml of dilution buffer, 10 mM Tris-HCL pH 7.4, 0.15 M NaCl) and filtered through 0.45 μm spin filters. Diluted samples (0.625 mL) were loaded at flow rate 0.5 mL/minute and flow-through fractions were collected and then concentrated using Amicon Ultra-15 centrifugal filters (Millipore, Billerica, MA). Eluted fractions were then obtained with 0.1 M glycine-HCL pH 2.5, neutralized with 0.1 M Tris-HCl pH 8.0, equilibrated with 10 mM Tris-HCL pH 7.4, 0.15 M NaCl at a flow rate 2 mL/min and saved for further studies.

### SELDI-TOF

Protein signatures of individual sera samples were performed by SELDI-TOF ProteinChip^® ^assays (Bio-Rad, Hercules, CA). The chip type selection (WCX2) and washing conditions were previously optimized [37]. Briefly, each chip was pretreated with 10 mM HCl, rinsed with HPLC grade water, then equilibrated with binding buffer (100 mM ammonium acetate, pH 4.0, with 0.1% Triton X-100). Each sample was diluted in binding buffer at a concentration of 0.02 ug/uL, with a total of 1 ug applied to each spot and incubated at room temperature for 30 min while shaking. Unbound proteins were removed by washing spots twice with binding buffer followed by washing with HPLC grade water. After drying each spot, 50% sinapinic acid (SPA) matrix was added to each spot, air-dried, then reapplied. SPA was prepared as a saturated solution containing 30% acetonitrile (ACN), 15% isopropanol, 0.5% trifluoroacetic acid (TFA) and 0.05% Triton X-100. The ionized proteins and their molecular mass/charge (m/z) ratios were detected using SELDI-TOF. The mass spectra were collected using a PBS II ProteinChip^® ^Biosystems and analyzed with ProteinChip^® ^software 3.2.1 (both from BioRad). The ProteinChip^® ^analysis was performed in triplicate. The ProteinChip^® ^reader was externally calibrated for each analysis using the four standard proteins: bovine insulin (5,733.6 Da), cytochrome C (12,230.9 Da), superoxide dismutase (SOD) (15,5941.4 Da), and β-lactoglobulin (18,636.3 Da). Peaks were automatically detected using Biomarker Wizard of ProteinChip^® ^software 3.2.1 with the following parameters: first-pass signal/noise (S/N) ratio = 5, second-pass S/N ratio = 2, and mass tolerance = 0.5%; estimated peaks were included in completion of clustering.

### HPLC (second dimension fractionation)

HPLC protein fractionation was performed using a liquid chromatography system (Shimadzu, Columbia, MD), which included a pump, system controller, manual injector with 500 uL injection loop, ultraviolet-visual (UV-Vis) detector set at 220 nm and fraction collector. The system was controlled using a Dell computer and EZStart chromatographic software (Shimadzu). The mobile phase consisted of mobile phase A (0.1 M ammonium acetate, pH 4.0) and mobile phase B (0.1 M ammonium acetate + 0.5 M NaCl, pH 4.0). A weak cation exchange column (100 × 2.1 mm; Eprogen, Darien, IL) was used for chromatographic separations of sera samples. The flow rate was set at 0.200 mL/min. Before sample injection, the column was pretreated with 10 mM HCl for 20 min and equilibrated with mobile phase A for 15 min. After sample injection, mobile phase A was continued for 30 min, then switched to mobile phase B for 30 min to 60 min. A total of 300 ug of each group (ND or HAD) in mobile phase A was injected into the column at time 0 min. Under these chromatographic conditions, bound proteins were eluted from the column between 40–45 min.

### 1-Dimensional Electrophoresis (1DE) (third dimension fractionation) and In Gel Tryptic Digest

WCX-HPLC fractions were mixed with NuPAGE^® ^LDS sample buffer (Invitrogen) and separated by 1DE. For this study, 30 ug of each fraction was loaded into each well of a NuPAGE^® ^Novex 12% Bis-Tris gel (Invitrogen) under reducing conditions using MES running buffer. Twenty micrograms of the WCX-HPLC fractions were vortexed in Novex^® ^Tricine SDS sample buffer (Invitrogen), loaded on to a 16% Novex^® ^gel and ran under reducing conditions using Tricine SDS running buffer. After electrophoresis, the above gels were stained with Sypro Ruby, followed by brilliant blue G-colloidal stain concentrate (Sigma) and protein bands were excised using a razor blade. After destaining with 100 uL of 50% ACN, 50 mM NH_4_HCO_3_/50% ACN and 10 mM NH_4_HCO_3_/50% ACN, the gel slices were dried and incubated with 0.1 ug/uL trypsin (Promega, Madison, WI) overnight at 37°C. Peptides were extracted with 0.1% TFA/60% ACN, dried and re-suspended in 0.5% TFA.

### Protein Identification

After tryptic digest, samples were purified using reverse phase C_18 _Zip Tips^® ^(Millipore, Billerica, MA) according to manufacturer's procedure and re-suspended in 0.1% formic acid in water prior to LC-MS/MS analysis. Protein identification was performed as described previously (Rozek et al 2007) using ESI-LC-MS/MS system (LTQ-Orbitrap, Thermo Scientific, Inc., San Jose, CA) in a nano-spray configuration using a microcapillary RP-C_18 _column (New Objectives, Woburn, MA) for fractionation. The spectra were searched using Sequest™search engine in BioWorks 3.2 software (Thermo Scientific Inc., San Jose, CA) using the following parameters: threshold for Dta generation = 10000, precursor ion mass tolerance = 1.4, peptide tolerance = 2.00 and fragment ions tolerance = 1.00. Database NCBI.fasta from  was used with two missed cleavage sites allowed and at least two peptides were required for protein identification.

### Western Blot Assays

1DE was performed on 7 HAD and 7 ND sera samples using NuPAGE gel system (Invitrogen Corp., Carlsbad, CA) in 4–12% gradient Bis-Tris gels under reducing conditions. For Western blot analyses, 2 μg of serum protein immunodepleted on an IgY column were loaded per lane. The gel was transferred to Immun-Blot PVDF transfer membrane using Ready Gel™Blotting Sandwiches (Bio-Rad, Hercules, CA). After blocking with 5% rabbit serum in PBST, the membrane was incubated with mouse anti-gelsolin antibody (BD Transduction Laboratories, San Jose, CA) followed by incubation with horseradish peroxidase-conjugated goat anti-mouse IgG (Jackson ImmunoResearch, West Grove, PA). A chemiluminescent signal was detected using SuperSignal West Pico™Chemiluminescent Substrate (Pierce, Rockford, IL) and signal was recorded on Blue Lite X-ray film (ISCBioExpress, Kaysville, UT). Images were scanned into Adobe Photoshop, adjusted using "Auto levels" and then analyzed using ImageJ software available through NIH. Image was inverted and a measurement box of exact same size was used for each band analysis. All numbers were exported into Microsoft Excel and measurements were normalized between membranes.

### Statistical Analysis

For SELDI-TOF data analysis, data from Biomarker Wizard were exported for statistical analysis using SAS^® ^software 9.1 (SAS Institute Inc., Cary, NC). Generalized estimating equations (GEE) were used to identify peaks that showed statistically significant differences in the distribution of intensity scores among the various replicates of HIV-infected individuals with or without HAD. The raw intensity values were found to be asymmetrical and transformed prior to analysis using the arsinh function: Y = log_2_(X + SQRT [X**2 + 1]), where 'X' is the observed intensity. This transformation has been used previously to stabilize intensity variance and make data more normally distributed and it has the advantage over a log-transformation of being able to transform negative values [21]. After the GEE modeling, the Bonferroni correction was used to address the issue of multiple testing.

## Competing interests

The authors declare that they have no competing interests.

## Authors' contributions

JW has made substantial contributions to data acquisition and analysis. Has been involved in drafting the manuscript revising it critically for important intellectual content. WR has made substantial contributions to data acquisition and analysis. WR has made substantial contributions to conception and design. Has been involved in revising manuscript critically for important intellectual content. FD has been involved in data analysis for this study. Has been involved in drafting the manuscript and revising it critically for important intellectual content. PC has made substantial contributions to conception and design, data analysis and interpretation. Has been involved in drafting the manuscript and revising it critically for important intellectual content. All authors have read and approved the final manuscript.

## Supplementary Material

Additional file 1 **Table S1. **Twenty one SELDI-TOF protein peaks showing statistically significant differences of intensities in sera samples from HIV-affected individuals with HAD compared to without HAD. Click here for file
