# Supplementary material for: Biomarkers of HIV-1 associated dementia: proteomic investigation of sera
Source: Proteome Sci. 2009 Mar 17;7:8. doi: 10.1186/1477-5956-7-8 (PMC2666658; doi:10.1186/1477-5956-7-8)
Supplement: Additional file 1 — Table S1. Twenty one SELDI-TOF protein peaks showing statistically significant differences of intensities in sera samples from HIV-affected individuals with HAD compared to without HAD. [file 1477-5956-7-8-S1.docx]

### Table S1. Twenty one SELDI-TOF protein peaks showing statistically significant differences of intensities in sera samples from HIV-affected individuals with HAD compared to without HAD.

| **Peak and Contrast** | **Median/Mean/SD (m/z)** | **Est. Intensity difference (transformed scale)** | **P- value*** | **Mean/SD of Intensity (natural scale)** | |
| --- | --- | --- | --- | --- | --- |
|  |  |  |  | **HAD** | **ND** |
| HAD vs ND | 4208/4208/1.71 | -0.7214 | 0.0055 | 2.599/1.7832 | 5.205/4.4281 |
| HAD vs ND | 4219/4219/1.31 | -0.4616 | 0.0175 | 1.836/1.0488 | 3.281/3.5590 |
| HAD vs ND | 4262/4262/0.92 | 0.5673 | 0.0227 | 3.237/4.6131 | 1.402/0.8117 |
| HAD vs ND | 4275/4275/1.60 | 0.5593 | 0.0361 | 5.007/3.7967 | 3.737/4.2493 |
| HAD vs ND | 4305/4305/1.55 | -0.3213 | 0.0496 | 2.252/1.9290 | 3.204/3.0995 |
| HAD vs ND | 4470/4471/1.47 | 0.3443 | 0.0492 | 3.666/3.0034 | 2.599/1.7486 |
| HAD vs ND | 4493/4493/1.05 | 0.4141 | 0.000368* | 1.957/1.6005 | 1.254/1.0093 |
| HAD vs ND | 4538/4537/1.64 | 0.3404 | 0.011 | 1.401/1.0705 | 0.987/0.8651 |
| HAD vs ND | 4649/4649/1.33 | 0.2821 | 0.0248 | 1.666/1.3015 | 1.225/0.9437 |
| HAD vs ND | 5175/5175/1.03 | -0.5061 | 0.0193 | 0.335/0.3967 | 1.076/1.8614 |
| HAD vs ND | 6420/6421/2.56 | -1.1146 | 0.00837 | 2.114/1.9522 | 6.654/8.0778 |
| HAD vs ND | 6435/6435/1.28 | -0.8697 | 0.000196* | 0.784/0.6645 | 2.242/2.5430 |
| HAD vs ND | 6576/6576/2.56 | -0.8357 | 0.0382 | 1.978/2.0277 | 4.458/5.4687 |
| HAD vs ND | 6591/6591/0.87 | -0.5408 | 0.0158 | 0.62/0.6538 | 1.349/1.5673 |
| HAD vs ND | 6633/6632/1.55 | -0.5253 | 7.80E-05* | 0.426/0.3212 | 0.938/0.7015 |
| HAD vs ND | 9290/9290/1.25 | 0.1043 | 0.00994 | 0.363/0.2709 | 0.28/0.1973 |
| HAD vs ND | 10635/10636/3.91 | -0.4453 | 0.0128 | 0.389/0.3162 | 0.893/0.9946 |
| HAD vs ND | 13523/13523/2.96 | -0.131 | 0.0189 | 0.288/0.1376 | 0.388/0.1999 |
| HAD vs ND | 17172/17172/3.88 | 0.101 | 0.00479 | 0.398/0.1230 | 0.324/0.1194 |
| HAD vs ND | 25646/25647/6.61 | 0.1789 | 0.0436 | 1.956/0.5437 | 1.7/0.5326 |
| HAD vs ND | 25872/25873/5.06 | 0.2861 | 3.79E-06* | 1.538/0.3937 | 1.201/0.3470 |

*:The peaks with P-value ≤ 0.001 (i.e., 0.05/50, criteria value after Bonferroni correction for multiple testing).
